# Examination of Lipoprotein and Lipid Levels of Adult Patients With Atherosclerosis, Myocardial Infarction, and Stroke: A Narrative Review

**DOI:** 10.7759/cureus.71239

**Published:** 2024-10-11

**Authors:** Nicholas Dushenko, Mykhailo Vysochyn

**Affiliations:** 1 Medicine, Saint James School of Medicine, The Valley, AIA; 2 Cardiology, Saint James School of Medicine, The Valley, AIA

**Keywords:** atherosclerosis, cholesterol, ischemic stroke, lipids, lipoproteins, myocardial infarction

## Abstract

The literature revealed correlations between high-density lipoprotein (HDL), low-density lipoprotein (LDL), and very low-density lipoprotein (VLDL) and vaso-occlusive disease. Specifically, positive linear relationships exist between LDL and VLDL and the development of vaso-occlusive disease. Alternatively, a U-shaped relationship between HDL and vaso-occlusive disease exists where both low and high levels of HDL increase the risk of developing conditions such as myocardial infarction and atherosclerosis. These results align with the National Institute of Health’s lipoprotein targets. Recent literature postulates that the adverse effects associated with lipoproteins may be attributable to commonalities between the structures of HDL, LDL, and VLDL, such as apolipoprotein subunits and molecular sizes that are not commonly analyzed in clinical lab tests. Nonetheless, additional research remains ongoing to further understand the role of lipoproteins in atherogenesis and vaso-occlusive disease.

## Introduction and background

Introduction

High-density lipoprotein (HDL) and low-density lipoprotein (LDL) are often believed to play inverse and causal roles, respectively, in the development of atherosclerosis and conditions relating to vaso-occlusive pathology. Examples of such pathologies include myocardial infarction (MI) and ischemic stroke. This is due to the abundance of apolipoprotein B (apoB) contained within LDL molecules that interact with cell-surface receptors, mediating the transfer of cholesterol from circulation into the cells. LDL molecules that become oxidized interact with blood vessel endothelium and trigger an inflammatory cascade, leading to the development of a fatty plaque within vessel walls [1]. HDL molecules have demonstrated the opposite effect in having cholesterol efflux capacity where cholesterol is removed from endothelial cells and macrophages to be transferred into the liver and excreted in the form of bile salts [2]. However, there remains an ongoing scientific discourse regarding the relevance and importance of these factors. For example, the 2018 recommendation for low-density lipoprotein according to the American Heart Association is a total cholesterol level of 150 mg/dL and LDL cholesterol equal to or below 100 mg/dl [1]. Alternatively, the American Diabetes Association (ADA) recommends LDL-C levels below 100 mg/dl in patients with diabetes and below 70 mg/dl for patients with both diabetes and cardiovascular disease [3]. Further review of the scientific literature yields several studies that demonstrate a wide variety of results regarding the effects of specific lipoproteins on morbidity. The findings of these studies are mixed, and the purpose of this systematic review is to examine the literature to find the lipoprotein, cholesterol, and triglyceride (TG) levels in adults aged 18-80 years that correlate with atherosclerosis, myocardial infarction, and stroke. These independent variables were selected as they comprise the standard lipid panel blood test. We will compare the results of our study to the current lipoprotein and triglyceride level recommendations set by the American National Institute of Health [4] to determine if the recommendations are in accordance with the latest findings in the literature. This review aims to address the following question: do the American National Institute of Health's lipoprotein and triglyceride targets remain relevant in the prevention of vaso-occlusive events such as atherosclerosis, MI, and ischemic stroke for adults aged 18-80?

## Review

Methods

Scientific literature pertinent to the subject was explored across databases such as PubMed and Google Scholar, filtering for the latest studies. The search was confined to studies published in English. The search criteria encompassed the disease denomination (thrombotic events, atherosclerosis, myocardial infarction, stroke) and keywords about lipid panel markers (LDL, HDL, VLDL, cholesterol, triglycerides). Furthermore, a meticulous system of manual examination of the studies' sample sizes and reference lists was carried out to include those with the broadest/most general implications.

Selection and inclusion/exclusion criteria

**Figure 1:**
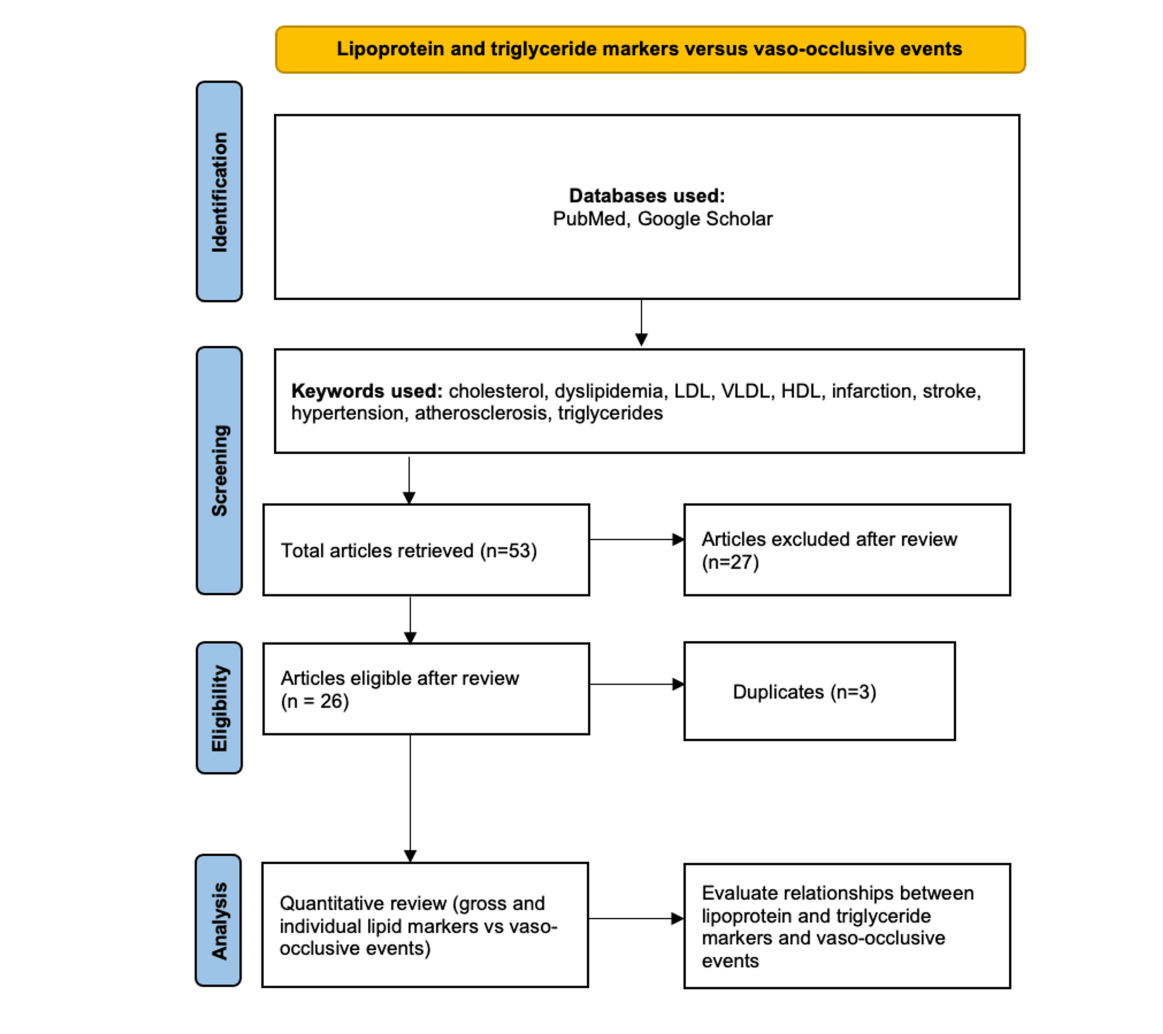
PRISMA flow chart illustrating the literature search PRISMA - Preferred Reporting Items for Systematic Reviews and Meta-Analysis

Incorporated into this review were studies that met specific inclusion criteria, which encompassed articles that focused on lipid markers in the context of vaso-occlusive pathologies, involved adults within the age range of 18-80, and scrutinized the relationship between levels of individual lipid markers and an apparent risk for specific conditions. Conversely, studies would be excluded if they involved a population that was too small or region-specific to have broader relevance or centered on investigations conducted on animal subjects. The abstracts underwent a rigorous assessment by two authors, and in instances where essential details were not ascertainable from the abstracts, the full articles were thoroughly reviewed. Figure 1 illustrates the search process in greater detail below.

Quality assessments

The studies that were initially amalgamated were briefly assessed by two authors who recorded any issues they had with their relevance or quality. There were no studies that were strictly excluded from the report based solely on either of these assessments, except for studies that did not end up meeting the inclusion criteria. This was carried out to examine the relative strengths and weaknesses of the studies and generate a more comprehensive final report.

Ethical considerations and acknowledgments

Since this study is a narrative review of published articles, it does not primarily involve human subjects and does not require ethical approval. The authors of this review have no financial incentives or conflicts of interest to disclose. No artificial intelligence tools were utilized in the preparation of this article. 

Results

A comprehensive manual literature search yielded 53 published articles published after the year 2000. After a two-step review process, 23 unique studies were included in the study and 27 studies did not meet the inclusion criteria to answer the research question. Three studies in the search were identified to be duplicate results. Included studies consisted of cross-sectional, case-control, narrative review, meta-analysis, retrospective cohort, and prospective cohort studies. All identified studies explored the following lipid markers: VLDL, LDL, HDL, total cholesterol, and triglycerides. Relevant details of the included studies are outlined in Table 1 below.

**Table 1:** Summary of identified studies LDL - low-density lipoprotein; HDL - high-density lipoprotein; VLDL - very low-density lipoprotein; TG - triglyceride; CVD - cardiovascular disease; MI - myocardial infarction

Study citation	Study design	Sample size	Age Range	Study Duration	Country	Molecules Analyzed	Results
Abdullah et al., 2018 [5]	Prospective study	36,375	18+	1978-1998	USA	LDL	Increasing levels of LDL increase risk of coronary heart disease and CVD mortality. LDL above or equal to 100 is associated with 30%+ increase in relative risk. LDL above or equal to 130 is associated with a 50%+ increase in relative risk. Non-HDL above or equal to 160 mg/dL was associated with CVD.
Alam et al., 2021 [6]	Case-control	131	41+	N/A	Bangladesh	Total Cholesterol, LDL, TG	Coronary heart disease cases demonstrated significantly elevated mean total cholesterol, LDL, and TG levels compared to controls.
Almahmoud et al., 2023 [7]	Retrospective cohort	485	60+	2010	Saudi Arabia	HDL	HDL levels below 20 mg/dL was associated with an increased risk of ischemic heart disease and all-cause mortality (hazard ratio 2.023, confidence interval 1.21-3.38).
Balling et al., 2023 [8]	Prospective cohort	68,289	N/A	2003-2015	Denmark	LDL	Elevations in LDL were significantly associated with an increased risk of atherosclerotic cardiovascular disease. Increases in LDL levels by 9 mg/dL increased risk for MI, ischemic stroke, and peripheral artery disease (hazard ratios were 1.26 for atherosclerotic cardiovascular disease, 1.28 for myocardial infarction and 1.22 for ischemic stroke).
Dong et al., 2021 [9]	Prospective Cohort	4128	24-96	2009-2020	China	Total Cholesterol	Total cholesterol below 155 mg/dL and above 185 mg/dL were significantly associated with increased risk of CHD and CVD (hazard ratios 1.933 and 1.561, respectively).
Gulec & Erol, 2020 [10]	Narrative Review	N/A	N/A	N/A	N/A	HDL	HDL-C levels above 90mg/dL (hazard ratio 1.36 for men above 97mg/dL, 2.06 when above 116mg/dL, 1.10 for women above 116mg/dL); and below 40mg/dL (hazard ratios approximately at 2.0 for men and women) demonstrate a significant hazard ratio indicating a potential increased risk for atherosclerotic cardiovascular disease.
Gu et al., 2019 [11]	Meta-analysis	267500	20+	1970-2008	China	Total Cholesterol, LDL, HDL, TG	Linear relationships were identified between total cholesterol, LDL and ischemic stroke. HDL and triglycerides demonstrated a non-linear relationship with ischemic stroke. Mean total cholesterol levels in patients with stroke were 190mg/dL compared to 182 for patients without stroke. For LDL, results were 109 mg/dL vs 104 mg/dL. The hazard ratio for HDL concentration and development of ischemic stroke showed a negative correlation relationship until 60mg/dL.
Haddad et al., 2002 [12]	Cross-sectional	354	30-76	2002	Jordan	Total Cholesterol, LDL, HDL, TG	Mean cholesterol, LDL and TG levels were elevated in patients diagnosed with CAD compared to controls (231 md/dL vs 202, 146 vs 118. 246 vs 164). Mean HDL levels were lower in patients with CAD compared to controls (36 mg/dL vs 44).
Heidemann, 2020 [13]	Prospective cohort	8139	18-80	1996-2017	Netherlands	VLDL	Elevated VLDL levels demonstrated significant hazard ratios in the highest interquartile ranges of VLDL to predict risk of developing major adverse limb events and MI in patients with existing cardiovascular disease. Non-HDL cholesterol levels can be useful in clinical practice, especially for patients at risk for adverse events despite low LDL.
Hsu et al., 2021 [14]	Prospective cohort	4072	20+	2002-2007	Taiwan	TG, LDL, non-HDL, apoB	Strong positive correlations were identified between baseline triglyceride and cholesterol levels and cardiovascular disease ranging from 0.78-0.91.
Ianuzzi et al., 2022 [15]	Cross-sectional	229	30-69	N/A	Italy	VLDL	Mean values of non-HDL-C/HDL-C ratio are significantly associated with intima-media thickness, indicative of atherosclerosis development.
Johansen et al., 2021 [16]	Prospective cohort	29,039	N/A	2003-2015	Denmark	LDL, VLDL	Individuals with elevated levels of VLDL and LDL demonstrated an elevated hazard ratio (3.5 and 1.3, respectively) for developing MI and atherosclerotic cardiovascular disease.
Kaneko et al., 2020 [17]	Retrospective cohort	1,451,997	20-49	N/A	Japan	LDL, HDL, TG	LDL>140, HDL <40 and TG >150 are independently associated with increased incidence of MI, angina and heart failure.
Khan et al., 2013 [18]	Prospective study	121	40-71	N/A	Saudi Arabia	TC, LDL, HDL, TG	Reduction in serum cholesterol does not prevent the risk of MI. HDL levels were inversely correlated with MI risk. Serum TGs did not significantly differ between MI patients and control.
Kumar & Das, 2018 [19]	Case-control	180	41-70	2017	India	TG, HDL, Total Cholesterol	Cases with coronary heart disease demonstrated significantly elevated levels of total cholesterol and triglycerides when compared to healthy controls. The cases also demonstrated significantly decreased HDL levels when compared to controls.
Liu et al., 2022 [20]	Prospective cohort	415,416	18+	1981-2016	England, Wales, Scotland	HDL	HDL levels greater than 80mg/dL (hazard ratio 1.11) and below 40 mg/dL increases risk of cardiovascular death independent of traditional risk factors (diabetes, MI history, BMI).
Liu et al., 2006 [21]	Prospective cohort	5794	30-79	1970-1994	USA	TG, LDL, HDL, VLDL	Non-HDL cholesterol was better at predicting coronary heart disease risk than LDL alone.
Mal et al., 2019 [22]	Case-control	421	N/A	2019	Pakistan	LDL, TC, HDL	Mean LDL levels were higher in those with MI compared to those without MI (95mg/dL vs 87 mg/dL), Mean HDL levels were lower in those with MI compared to those without MI (38 mg/dL vs 41 mg/dL). Mean total cholesterol in those with MI was 171 mg/dL vs 160 mg/dL in those without MI.
Ren et al., 2010 [23]	Prospective cohort	30,378	35-64	1992-2007	China	VLDL	The population-attributable risk proportion of CHD associated with VLDL cholesterol was 17.3% higher than that associated with LDL cholesterol alone. Risks were elevated when both VLDL and LDL were elevated.
Schubert et al., 2023 [24]	Prospective cohort	63,168	18+	2006-2016	Sweden	LDL	Individuals with LDL levels below 43 mg/dL experienced a reduced risk for MI compared to individuals with LDL 64 mg/dL or above (hazard ratio 1.16).
Tarchalski et al., 2003 [25]	Prospective cohort	141	45-61	2003	Poland	LDL, TG, HDL	LDL and TG levels were positively correlated with the extent of coronary atherosclerosis in patients. HDL was negatively correlated with coronary atherosclerosis.
Togha et al., 2011 [26]	Cross-sectional	445	40-80	2006-2007	Iran	Total cholesterol, TG, HDL, LDL	LDL levels may influence the risk of developing ischemic stroke. Patients presenting with ischemic stroke demonstrated significantly elevated mean LDL values compared to controls (124 mg/dL vs 147 mg/dL).
Willey et al., 2009 [27]	Prospective cohort	2940	39+	1993-2001	USA	HDL, LDL, total cholesterol, TG	Only LDL levels demonstrated a significant relationship with ischemic stroke. LDL > 130mg/dL showed an increased risk with a hazard ratio of 3.81 (confidence interval 1.53-9.51).

The studies cited by this narrative review identified a variety of relationships between lipid markers and vaso-occlusive events. Generally, increasing levels of LDL, VLDL, total cholesterol, and triglycerides were associated with increased risks of adverse events such as myocardial infarction, progression of atherosclerosis, and ischemic stroke. Statistically significant metrics, such as positive correlation coefficients, incidence rates, hazard ratios, and risk ratios supporting these relationships, were identified by the authors of the studies reviewed in this report, as noted in Table 1. One study by Dong et al. [9] deviates from this pattern in its finding that total cholesterol levels below 155mg/dL and above 185 mg/dL both increase the risk of developing coronary heart disease and cardiovascular disease (p<0.05 with adjusted hazard ratios of 1.933 and 1.502, respectively). The authors mention that only a relatively small proportion of the study's population sample from rural China had total cholesterol values below 155mg/dL, offering a potential explanation for the wide confidence interval (1.248-2.993) and making it difficult to generalize the claim regarding increased vaso-occlusive risks to the general population of those in China and the developed world. Interestingly, this relationship did not exist with HDL cholesterol. HDL cholesterol was shown to have a cardio-protective effect when maintained within optimal levels, suggesting that a "U-shaped" relationship exists where a lower and upper boundary exists to achieve its beneficial effects and minimize the risk for experiencing vaso-occlusive disease. This finding was revealed by the studies with the largest sample sizes, as it is unusual for individuals to have high HDL values, challenging the idea that the relationship between HDL and vaso-occlusive events follows a predictable negative correlation.

Discussion

The findings of this narrative review support the target lipoprotein, cholesterol, and triglyceride targets recommended by the American National Institute of Health (tables 2 & 3). The results of the studies used in this narrative review demonstrate positive correlations in risk for the vaso-occlusive events of interest and LDL, VLDL, triglycerides, and total cholesterol. This largely aligns with the stepwise elevations in risk outlined in Table 2 and Table 3 below, supporting the general principle that "lower is better" for mitigating the risk of developing atherosclerosis, MI, and ischemic stroke. This observation also correlates with the differing recommendations for LDL targets set out by the American Heart Association and American Diabetes Association, as their targets are based on overall patient risk for the development of vaso-occlusive events. Patients with increased risk for the development of such pathological events are recommended to achieve stricter LDL targets. Since explicit values for VLDL are not available within the National Institute of Health's guidelines, explicit VLDL-level targets may be an area for authors of future clinical practice guidelines to explore. Furthermore, the U-shaped relationship between HDL and the vaso-occlusive events identified by the studies compiled within this narrative review is also demonstrated within the National Institute of Health's guidelines. Additional research regarding the mechanisms of atherogenic molecules such as LDL and VLDL remains ongoing, with studies indicating the potential of lesser-known but commonly shared components such as apolipoprotein B (apoB) as being substances that contribute to plaque formation [1,2,28,29].

**Table 2:** American National Institute of Health lipoprotein and cholesterol targets (units in mg/dL) LDL - low-density lipoprotein; HDL - high-density lipoprotein Source: Abdullah et al. [5]

LDL cholesterol - primary target of therapy	
<100 mg/dL	Optimal
120-129 mg/dL	Near optimal/Above optimal
130-159 mg/dL	Borderline high
160-189 mg/dL	High
≥190 mg/dL	Very high
Total cholesterol	
<200 mg/dL	Desirable
200-239 mg/dL	Borderline high
≥240 mg/dL	High
HDL cholesterol	
<40 mg/dL	Low
≥60 mg/dL	High

**Table 3:** American National Institute of Health triglyceride targets (units in mg/dL) Source: Abdullah et al. [5]

<150 mg/dL	Normal
150-199 mg/dL	Borderline high
200-499 mg/dL	High
≥500 mg/dL	Very high

The current and future relevance of this research work to the authors of this narrative review remains high. Presently, the authors' common misconception that the relationship between the often-touted cardioprotective HDL and vaso-occlusive events is negatively correlated was challenged by the findings of increased risk of harm at high levels of HDL concentration. Additionally, the authors were able to gain an appreciation for the vast body of literature surrounding the research topic and the rigors of research necessary to formulate clinical practice guidelines in this modern era of evidence-based medicine. The knowledge acquired through the completion of this project will remain relevant as we transition to clinical practice, where we will encounter patients with varying levels of risk for vaso-oclusive events who rely upon our expertise to appropriately manage their care based on their risk levels.

Limitations

This narrative review has its limitations. First, the literature searches were only performed in two databases. Identified studies were limited to those published in the English language, and no grey literature was included. While the authors decided to narrow their search to studies published after the year 2000 to focus on the latest evidence regarding the research topic, relevant studies published prior to this year are widely available. Broadening the search strategy and inclusion criteria could add further support or alternative information to our study's collection of findings. To establish more precise findings, such as significant therapeutic target values for lipoproteins, a systematic review and meta-analysis of the included studies could be completed.

## Conclusions

There is substantial evidence that supports the lipoprotein targets outlined by the American National Institute of Health and the American Heart Association. The literature routinely demonstrates relationships between lipoprotein levels (HDL, LDL, VLDL, non-HDL) and the development of atherosclerosis, myocardial infarction, and ischemic stroke. Increases in LDL, VLDL, TGs, total cholesterol, and non-HDL levels positively correlate with the development of vaso-occlusive events, while HDL shows a U-shaped correlation where levels below or above the recommended limits are risk factors for pathological vascular events. The authors of this narrative review support the recommendations outlined by the American National Institute of Health found in Table 2 and Table 3. Based on these findings, it is evident that current lipoprotein targets are reasonable, and clinicians should work closely with their patients to set and achieve these goals through physical activity, dietary and/or pharmacological methods to reduce their progression of atherosclerosis, risk of MI, and ischemic stroke.
